# Synthesis, modification and graft polymerization of magnetic nano particles for PAH removal in contaminated water

**DOI:** 10.1186/2052-336X-12-105

**Published:** 2014-07-15

**Authors:** Azadeh Torabian, Homayoun Ahmad Panahi, Gholam Reza Nabi Bid Hendi, Naser Mehrdadi

**Affiliations:** 1 Department of Environmental Engineering, University of Tehran, Tehran, Iran; 2 Department of Chemistry, Islamic Azad University, Tehran, Iran

**Keywords:** Magnetic nano particles, Poly aromatic hydrocarbons, Nano adsorbents, Water contamination

## Abstract

Magnetic nanoparticles (MNPs) were modified with 3-Mercaptopropytrimethoxysiline (MPTMS) and grafted with allyl glycidyl ether for coupling with beta naphtol as a method to form a novel nano-adsorbent to remove two poly aromatic hydrocarbons (PAHs) from contaminated water. The modified MNPs were characterized by transmission electron microscopy, infrared spectroscopy and thermogravimetric analysis. Results showed that the modified MNPs enhanced the process of adsorption. Tests were done on the adsorption capacity of the two PAHs on grafted MNPs; factors applied to the tests were temperature, contact time, pH, salinity and initial concentration of PAHs. Results revealed that adsorption equilibrium was achieved in 10 min, and the maximum adsorption capacity was determined as 4.15 mg/g at pH = 7.0 and 20°C. The equilibrium adsorption data of the two PAHs by the modified MNPs were analyzed by Langmuir, Freundlich and Temkin models. Equilibrium adsorption data was determined from the Langmuir, Freundlich and Temkin constants from tests under conditions of pH = 7 and temperature 20°C. Analysis of the adsorption-desorption process indicated that the modified MNPs had a high level of stability and good reusability. Magnetic separation in these tests was fast and this shows that the modified MNPs have great potential to be used as a new adsorbent for the two PAHs removal from contaminated water in water treatment.

## Introduction

Contamination generated by petroleum compounds has raised concern all over the world [1]. Petroleum compounds are a complex mixture of different hydrocarbons. Among the hydrocarbons present in petroleum, the category of polycyclic aromatic hydrocarbons (PAHs) is a very important source of water contamination. PAHs constitute hazardous organic chemicals that consist of two or more benzenoid groups. They are ubiquitous pollutants in our environment.

It is important to further our understanding of PAHs because of its potential carcinogenicity and mutagenicity [2]. Reports published by the Center for Children’s Environmental Health demonstrate that exposure to PAHs pollution during pregnancy can contribute to problems such as premature delivery and neonatal conditions of low birth weight and heart malformation. Heavy exposure to PAHs carries health risks to the lung and skin and bladder cancer [3].

Effluent from petrochemicals, petroleum refineries, and from continuous fuel leakage from underground gasoline storage tanks in urban areas with old and cracked storage tanks are the main sources of PAHs contamination in water and groundwater [1]. Accidents in the transportation of petroleum fuels and the fracture of old oil pipes also contribute to the release of PAHs into the environment [4-6]. Advanced technology for measuring environmental contamination in water sources, coupled with recent developments in health science has determined restrictions and set limits for levels of these compounds in water. The permitted concentration of PAHs in drinking water is limited to is 3 mg/L [7].

Recent developments in nanotechnology and its increasingly widespread application, particularly in the use of nano particles (NPs) in water and wastewater treatment, makes using NPs as adsorbents interesting. Recently using the magnetic effect of a certain type of nano particle has been applied to ease the process of separating, removing/isolating particular components from a sample solution. Properties of nano sized magnetic iron oxide particles such as having a large surface area and low level diffusion resistance [8].

Research has been done on the separation and removal of chemical species such as metals [8-14]; dyes [9,15,16] and gases [8]. Considerable attention has been paid to the combination of organic groups and inorganic magnetic Fe_3_O_4_ particles at the nano-sized level for its high specific surface area with an absence of internal diffusion resistance in comparison to traditional micron-sized support particles [17,18]. Meanwhile, magnetic nano-sized carriers are easily separated from solutions by use of an external magnetic field [19].

Many methods have recently been used for the production and modification of Fe_3_O_4_ NPs; these methods include co-precipitation, microemulsion, thermal decomposition and hydrothermal synthesis [20,21]. Co-precipitation is an easy and convenient method to synthesize iron oxides from aqueous Fe2+/Fe3+ salt solutions in the presence of a base, it produces a high yield and a relatively narrow size-distribution [22,23]. In general, the modification of synthesized NPs prevents particles from agglomerating and increases the interactivity between an absorbent and a specific contaminate [24].

In this study, MNPs were synthesized and grafted with a functional monomer. The grafted MNPs (GMNPs) showed a good adsorption capacity for removing two PAHs that were easily separated from the sample solution in the presence of an external magnetic field. The GMNP was characterized and then its adsorption capacity was determined. Tests were done on factors affecting adsorption; pH, salinity, initial concentration of PAHs, temperature and contact time. The method was successful in determining PAHs from an aqueous water sample.

## Materials and methods

### Instruments

Chromatographic separations were carried out on an Agilent HPLC, 1200 series, equipped with a UV/Vis detector. Separations were carried out on a Zorbax Extend C18 column (15 cm - 4.6 mm, with 3 mm particle size) from the Agilent Company (Wilmington, DE, USA). The acetonitrile at a flow rate of 2 ml/min, was used as a mobile phase in isocratic elution mode. The injection volume was 10 μL for all samples, and the detection was performed at a wavelength of 220 nm. The pH measurements were taken with a metrohm meter, model 744 (Zofingen, Switzerland). Infrared spectra were determined using a jasco fourier transform infrared spectrometer (FT-IR) model FT-IR400 (Maryland, USA). Thermogravimetric analysis (TGA) was made using the Shimadzou model TGA-50H (Kyoto, Japan). The samples were characterized with a transmission electron microscope (TEM) Model-JEM 2010 (Tokyo, Japan).

### Materials

**Figure 1:**
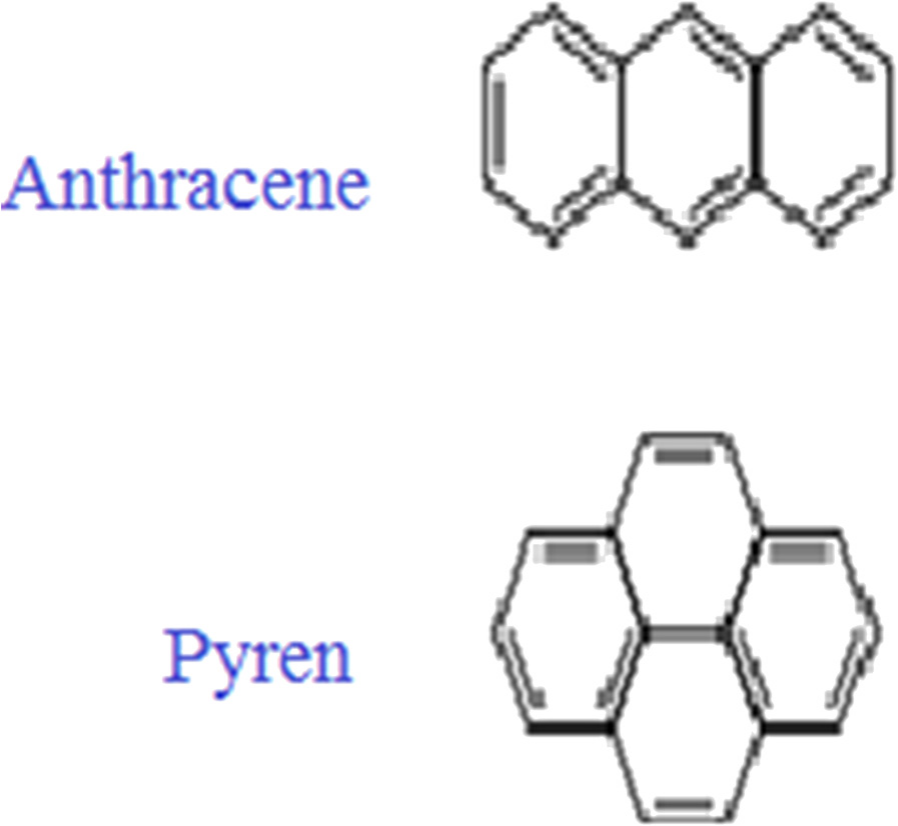
The molecular structure of ANT and PYR.

N, N-Dimethylformamide (DMF), 3-mercaptopropyltrimethoxysilane (MTPMS), allyl glycidyl ether (AGE) and 2, 2-azoisobutyronitrile (AIBN), were products of Aldrich (Steinheim, Germany). 1, 4-Dioxane, 2-naphtol, NaCl, C_2_H_5_OH, CH_3_COOH, FeCl_2_.4H_2_O, FeCl_3_.6H_2_O, NH_4_OH, C_14_H_10_, C_16_H_10_ were supplied by Merck (Darmstadt, Germany). Anthracen (ANT) and pyren (PYR) Figure 1, were purchased from Fluka Chemical (Buchs Switzerland). The molecular structure of ANT and PYR is shown in Figure 1.

All the reagents were of analytical grade and used without any further purification. Because of the low solubility of ANT and PYR the first stock solution (2000 mg/L) was prepared by dissolving appropriate amounts of ANT and PYR, in first acetonitrile and diluting with distilled water. The secondary stock solution (10 mg/L) was prepared by dilution. 10 mL of 0.1 M acetic acid–acetate buffer (pH 3–6.5) was used wherever required to adjust pH of the solution.

### Synthesis of GMNPs

#### Synthesis of MNPs

MNPs were prepared by chemical co-precipitation [25]. MNPs were prepared by adding 100 mL of distilled water into a three-necked 1000 mL bottle. Nitrogen gas was injected into the water for 30 mins. Subsequently 2.78 g of FeSO_4_ · 7H_2_O and 3.24 g of FeCl_3_ · 6H_2_O were first dissolved in 10 mL of distilled water and then added to the three-necked bottle under nitrogen atmosphere. Ammonia solution was added drop wise to the above solution under mechanical stirring [26]. The reaction was allowed to take place at 80°C for 2 h. During the process of adding ammonia, the color of the solution changed from its original brown color to dark black that indicated the formation of MNPs. A magnetic field was then applied to the external edge of the glass reactor. The black oxides responded to the magnetic force, proving the reaction was complete. The obtained MNP precipitate was separated and then washed twice with 500 mL deionized water.

### Modification of MNPs with MPTMS

The second step involved the modification of MNPs with MPTMS followed by grafting AGE on to the modified MNPs [27]. Before starting the silyation reaction, MNPs were cleaned with 1 M ethanol. The washed MNPs were dried for 24 h. At this point, 3 g of MNPs were silylated by an anhydrous solution of 5% of MPTMS in 47.5 mL of 1, 4-dioxane. The reaction was allowed to take place in a 1- necked round-bottom flask (equipped with a condenser) at boiling point of the solution for approximately 24 h. Eventually, the modified MNPs were washed several times with 1,4-dioxane and dried under a vacuum in a desiccator over dry calcium chloride.

### Graft polymerization of modified MNPs

The free radical graft polymerization of AGE onto MPTMS-modified MNPs was carried out in a temperature-controlled reactor under vigorous stirring in a nitrogen atmosphere. The modified MNPs with MPTMS were transferred into a degassed polymerization solution containing (20 mL ethanol, 20 mL AGE, and 0.02 mg AIBN) for 6 h at the temperature of 70°C. The grafted MNPs were separated by a magnetic field and washed with 100 mL of ethanol, water and then once more with ethanol, and then dried under vacuum conditions in a desiccator over dry calcium chloride.

### Coupling 2-Napftol to grafted –modified MNPS

**Figure 2:**
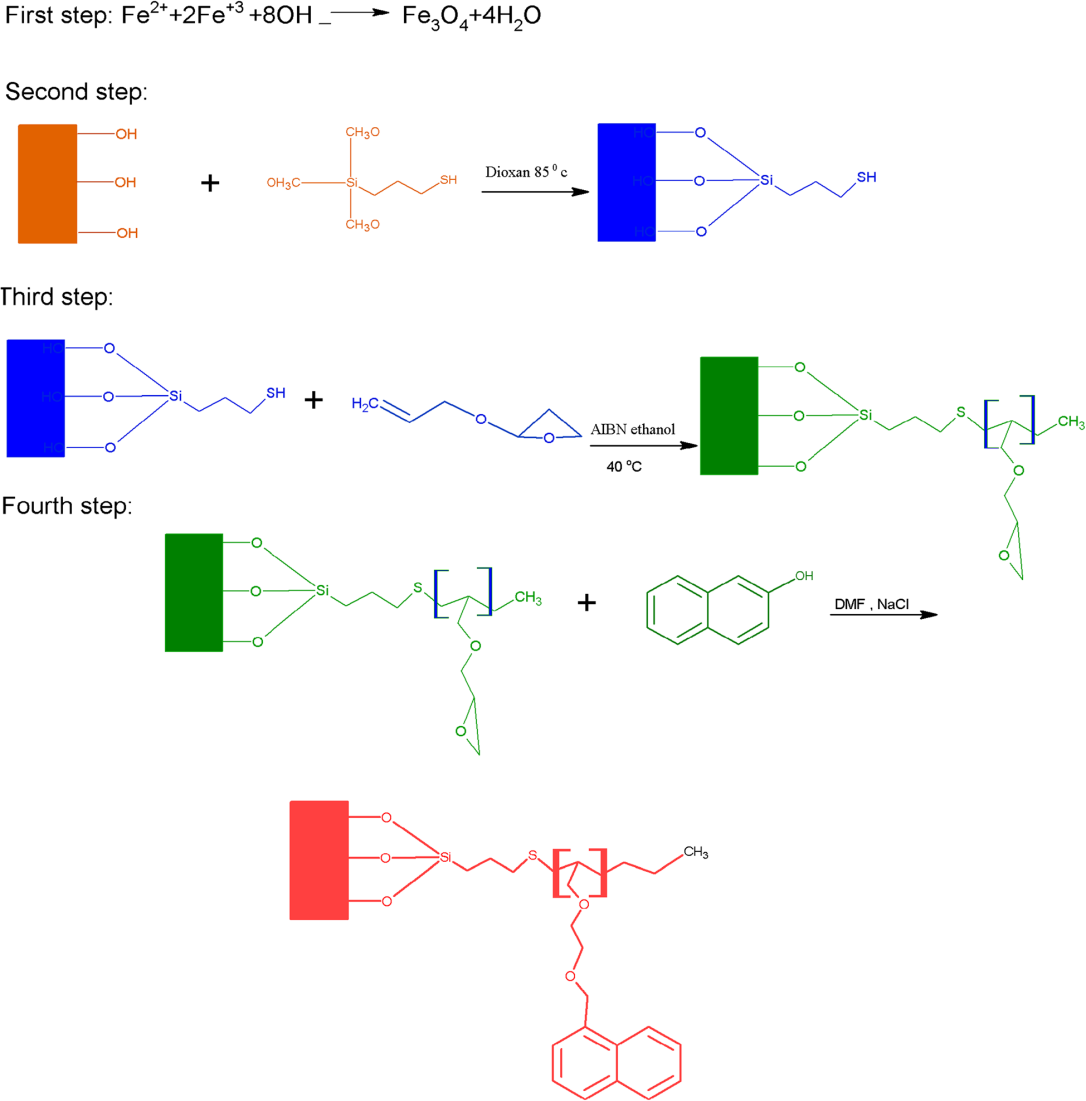
Schematic presentation of synthesis and the grafting – coupling process of MNP.

The final step was coupling 2-napftol on to the grafted and modified MNPs. The reaction took place by adding the grafted modified MNPs and 1 g of 2-naphtol dissolved in 40 mL DMF into a temperature-controlled reactor. The reaction was allowed 8 h to complete at room temperature. The coupled MNPs were separated by a magnetic field and then washed with 100 mL of ethanol, water and then ethanol once again and dried under a vacuum in a desiccator over dry calcium chloride.The aromatic rings of 2-naphtol enable adsorption by the reaction of π - π between PAHs and the coupled -grafted-modified MNPs (CGMMNPs). The complete procedure followed for this synthesis is shown in Figure 2.

The CGMMNPs were characterized by FT-IR, TGA, TEM.

### Batch method of PAHs adsorption

The adsorption of the two PAHs by the CGMMNPs (as a magnetic nano adsorbent) was studied using the technique of batch equilibrium in aqueous solutions. Evaluations were taken for various parameters that affect the removal of the PAHs and were studied and optimized by univariate method. In general, 0.02 g of GMNP was added to 5 mL of PAHs solution containing ANT/PYR (as an indicator of PAHs) of various concentrations and shaken in a thermostatic water-bath shaker. After 10 min, the magnetic nano-adsorbents were removed using a magnetic field and the remaining supernatant ANT/PYR concentrations were measured using HPLC (Agilent 1200 series). The amount of PAHs at equilibrium qe (mg/g) on CGMMNPs was calculated from the following equation.

(1) $$\mathrm{qe}\;=\;\left(C_{0}-\;\mathrm{Ce}\right)V/W$$

Where C_0_ and Ce (mg/ L) are the initial and equilibrium concentrations of PAHs, respectively, V (L) is the volume of the solution and W (g) is the mass of the adsorbent used.

## Results and discussion

### Characterization of CGMMNPs

**Figure 3:**
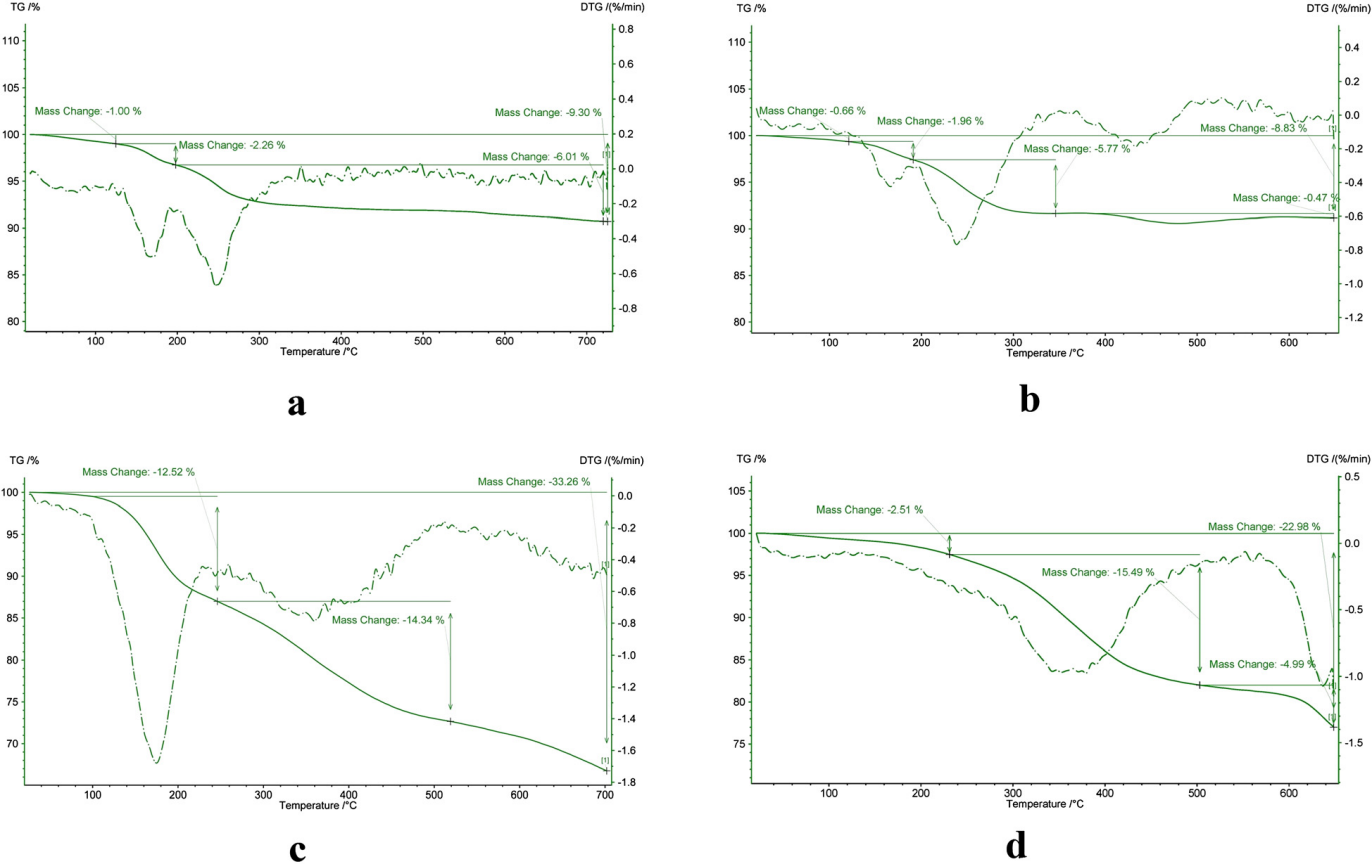
Thermogravimetric analysis of MNPS (a), modified MNPs (b), grafted MNPs (c) CGMMNPs (d).

**Figure 4:**
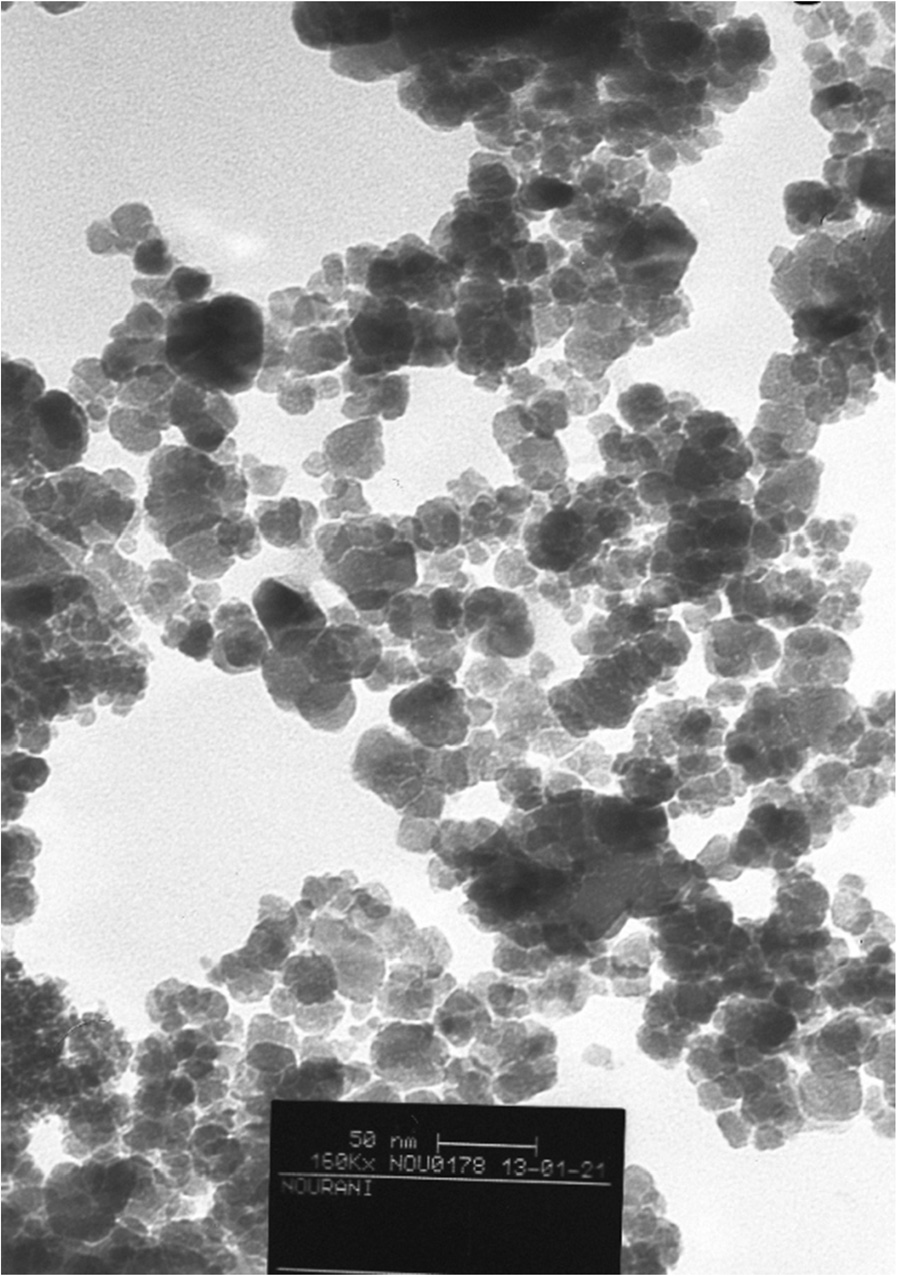
TEM photograph of CGMMNPs.

The CGMMNPs were characterized by FT-IR, TGA and TEM. The FT-IR spectrum of CGMMNPs was compared with raw MNPs as well as FT- IR; (NaCl, cm−1) 3779.8 (OH), 1627 (C=O), 1450 (aromatic cycle), 3051.53 (aromatic C-H) and 1000 (C–O). The presence of aromatic group by FT-IR spectrum of CGMMNPs indicates that the coupling of 2-naphtol was successful. The TGA of unmodified MNPs indicated a weight loss of up to 120°C, and this can be attributed to desorption of the water molecules from the surface at temperature higher than 200°C the weight remained constant. GMNPs however showed completely different thermal behavior. Weight loss up to the temperature of 200°C was due to the presence of water molecules in the grafted MNPs and weight loss at 220–500°C was caused by decomposition and desorption of the polymeric matrix Figure 3. These results demonstrate the formation of CGMMNPs. Briefly, FT-IR spectrum and TGA confirmed the structure of the grafted polymer as presented in Figure 2. TEM was used to examine the external surface of the CGMMNPs. As indicated in Figure 4 the particles were spherical with a rough surface and particle size was 15–40 nm.

### Adsorption properties of CGMMNPs for PAHs

#### Effect of pH on adsorption

**Figure 5:**
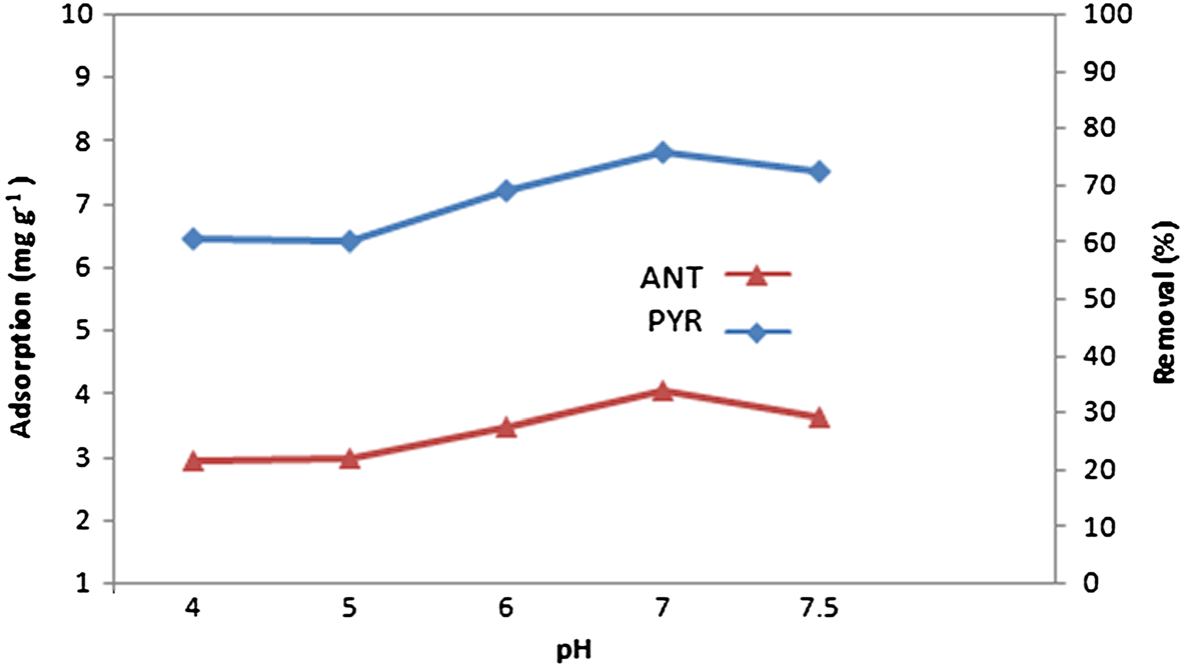
Effect of pH on adsorption of ANT/PYR on CGMMNPs.

The effect of pH was studied at the range of (4–8) with the initial concentration of 20 μg/ L. As shown in Figure 5 pH had a minor effect. A suitable pH value can improve adsorption efficiency and reduce matrix interference. The removal of ANT/PYR in solution decreased according to an increased pH of the aqueous solution from 4 to 5. The optimum pH for adsorption was 7, suggesting that a neutral pH was ideal for the adsorption process. This can be explained by the different solubility of ANT and PYR in various pH. No precipitation occurred at this pH level. A pH level above 8 was not considered because of the possibility for dissolving and decomposing MNPs.

### Effect of initial concentration of the PAHs on adsorption

**Figure 6:**
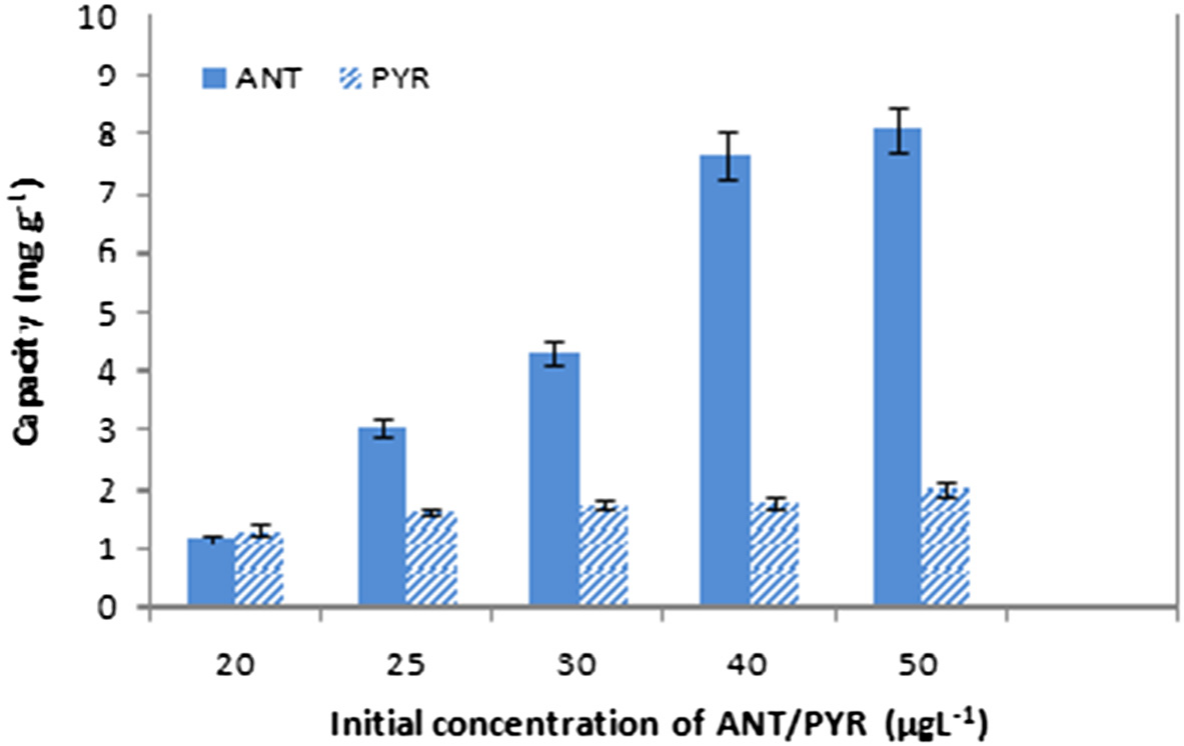
Effect of initial concentration of ANT/PYR adsorption on CGMMNPs.

Considering ANT and PYR maximum solubility and detection limits the experiments were done in the range of 20–50 μg/L. Results indicated that increasing the amount of PAHs, resulted in an increase in removal capacity Figure 6. An increase in the concentration of PAHs leads to more of the π - π interaction indicating an equilibrium reaction leading to more effective removal.

### Effect of contact time on adsorption

**Figure 7:**
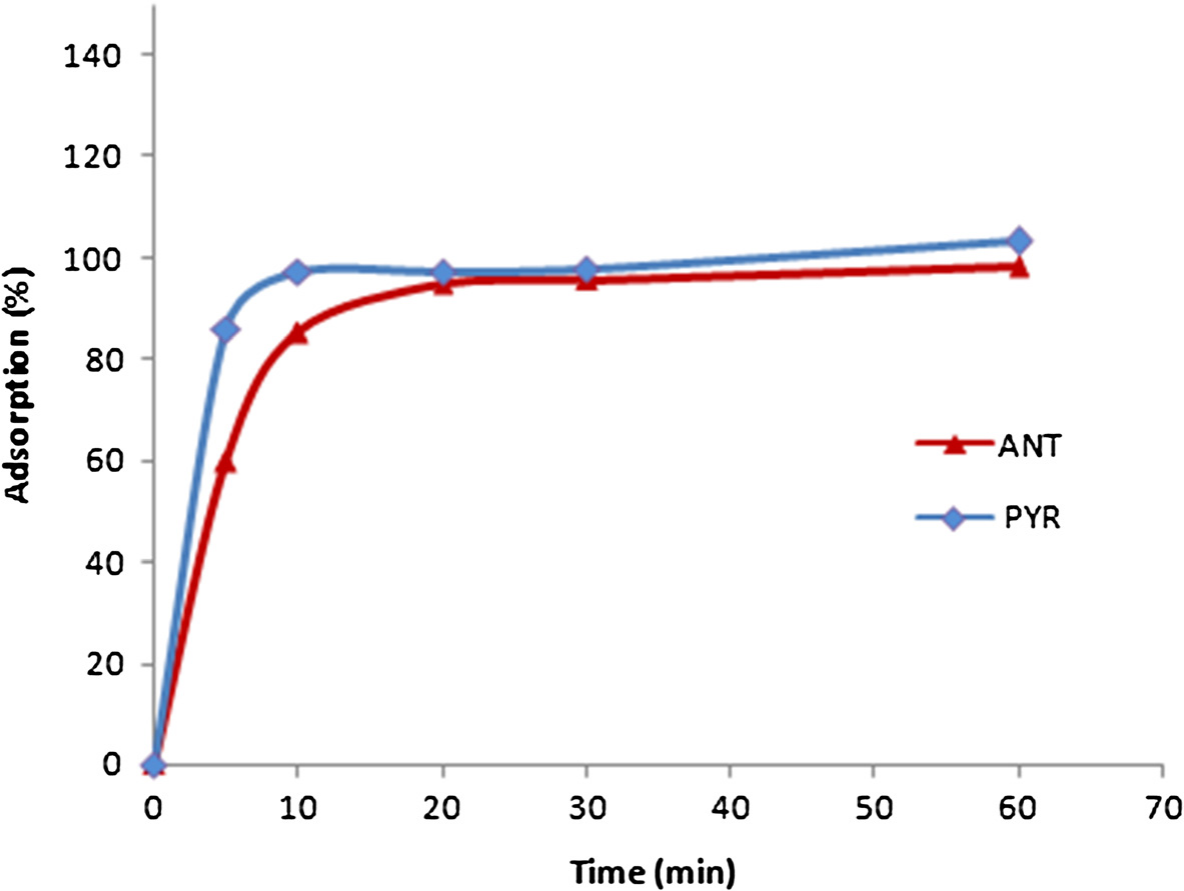
The kinetic study of removal ANT/PYR from solution.

The kinetic study of removal PAH from solution by the CGMMNPs showed that 10 minutes was sufficient for the complete sorption of ANT and PYR. The profile of ANT and PYR uptake by the CGMMNPs reflected good accessibility to active sites in the sorbent. Figure 7.

### Effect of temperature on adsorption

**Figure 8:**
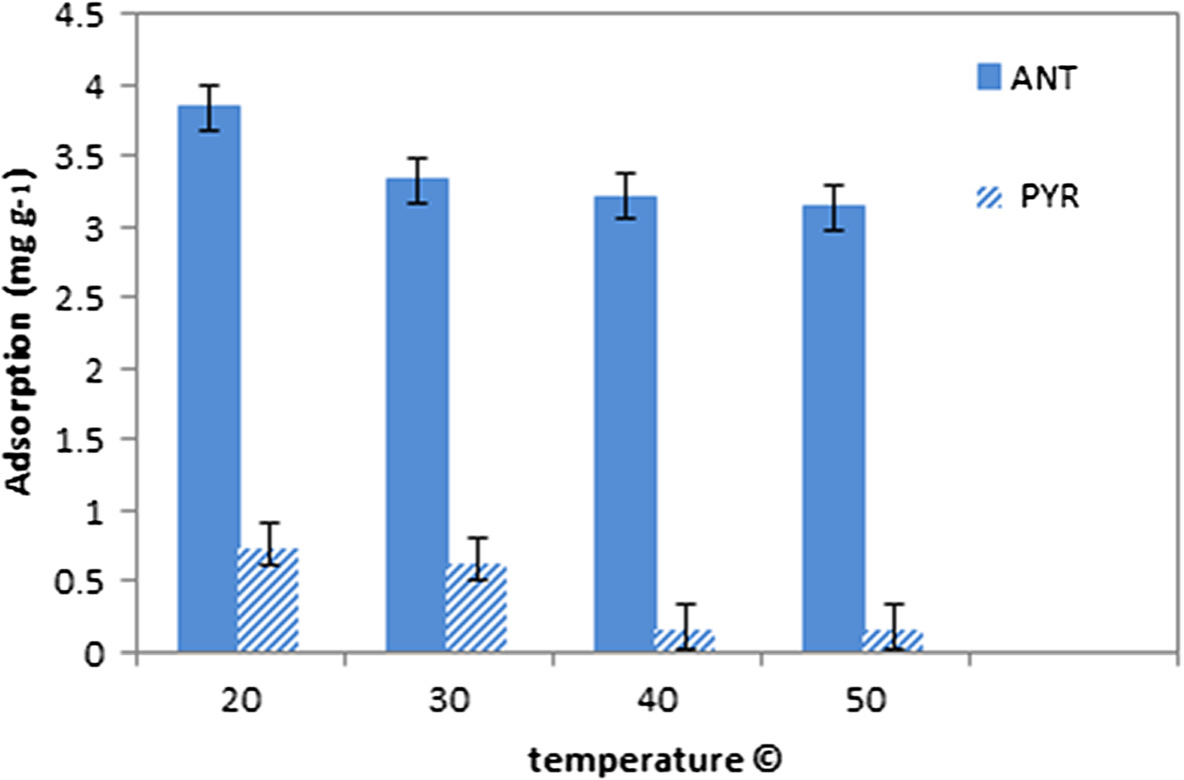
Effect of temperature on ANT/PYR adsorption by CGMMNPs.

The effect of different temperatures on ANT/PYR removal was investigated under optimized conditions determined by previous experiments as shown in Figure 8. High temperature had a negative effect on sorption due to increased molecular mobility. The optimum temperature was determined as 20°C and as such is advantageous because the reaction for removal of the PAHs by CGMMNPs can take place at room temperature.

### Effect of total dissolved solids (TDS) on adsorption

**Figure 9:**
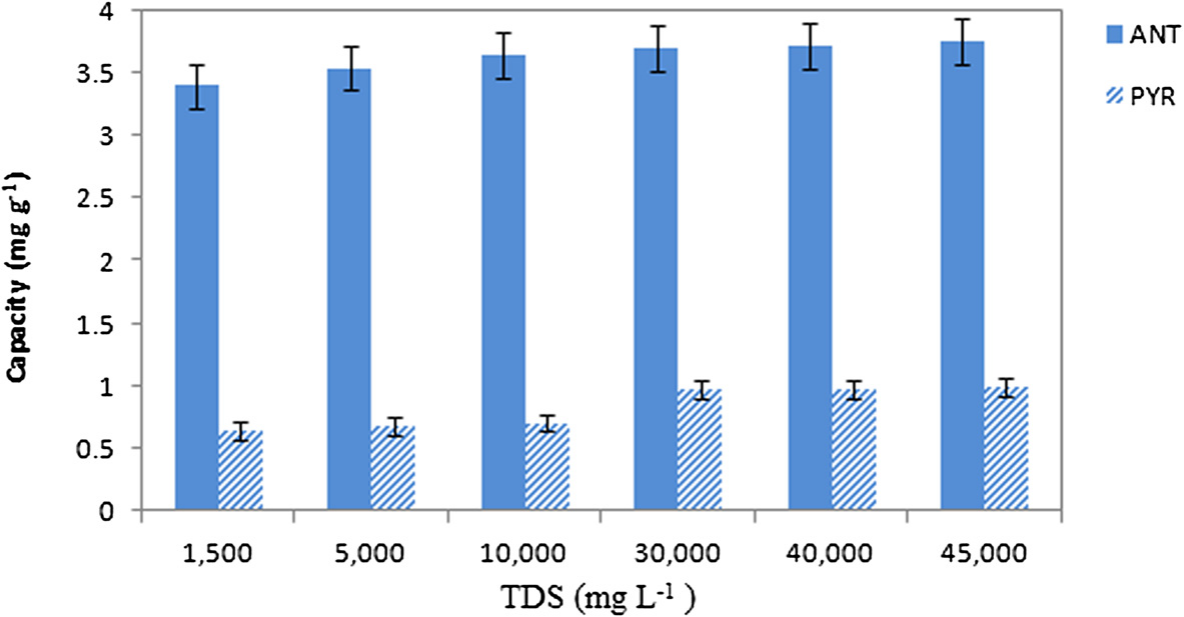
Effect of TDS on ANT/PYR adsorption on CGMMNPs.

The effect of TDS as an important parameter in water quality was illustrated by the removal of ANT/PYR under optimized conditions by Figure 9 NaCl ranging 0–3.5% was added to the solution for obtaining the required TDS. Increasing the percentage of NaCl improved the removal of PAHs due to the salting out effect.

### Adsorption isotherm studies

Equilibrium isotherms are used to describe and define adsorption data. By comparing collaboration with different presented models, surface modification and sorption mechanisms can be described in more detail according the following.

• Langmuir equation

(2) $$\mathrm{Ce}/\mathrm{qe}=\;\left(1/q_{\text{max}}.K_{L}\right)\;+\;\left(\mathrm{Ce}/q_{\text{max}}\right)$$

• Freundlich equation

(3) $$\ln\;q_{e}=\;\mathrm{Ln}\;K_{f}+\;1/n\;\ln\;C_{e}$$

• Temkin equation

(4) $$q_{e}=B\;\ln\;A\;+B\;\ln\;C_{e}$$

In the langmuir equation (2), Ce is described as the equilibrium PAHs concentration in the solution (mg/L), q_max_ is the maximum adsorption capacity relevant to surface coverage (mg/g), K_L_ the Langmuir constant (L/mg), qe is the concentration of PAHs on the adsorbent (mg/g).

In Freunlich the equation (3), qe and Ce are the same as described and K_f_ is the Freunlich constant (L/g), n is a dimensionless factor of heterogeneity.

In the Temikin equation (4), B=RT/b and b is the Temkin constant related to sorption heat (J/mol), R is the gas constant (8.314/ mol°K ), A is Temkin isotherm equilibrium binding constant (L/g), and T is the temperature (293°K).

**Figure 10:**
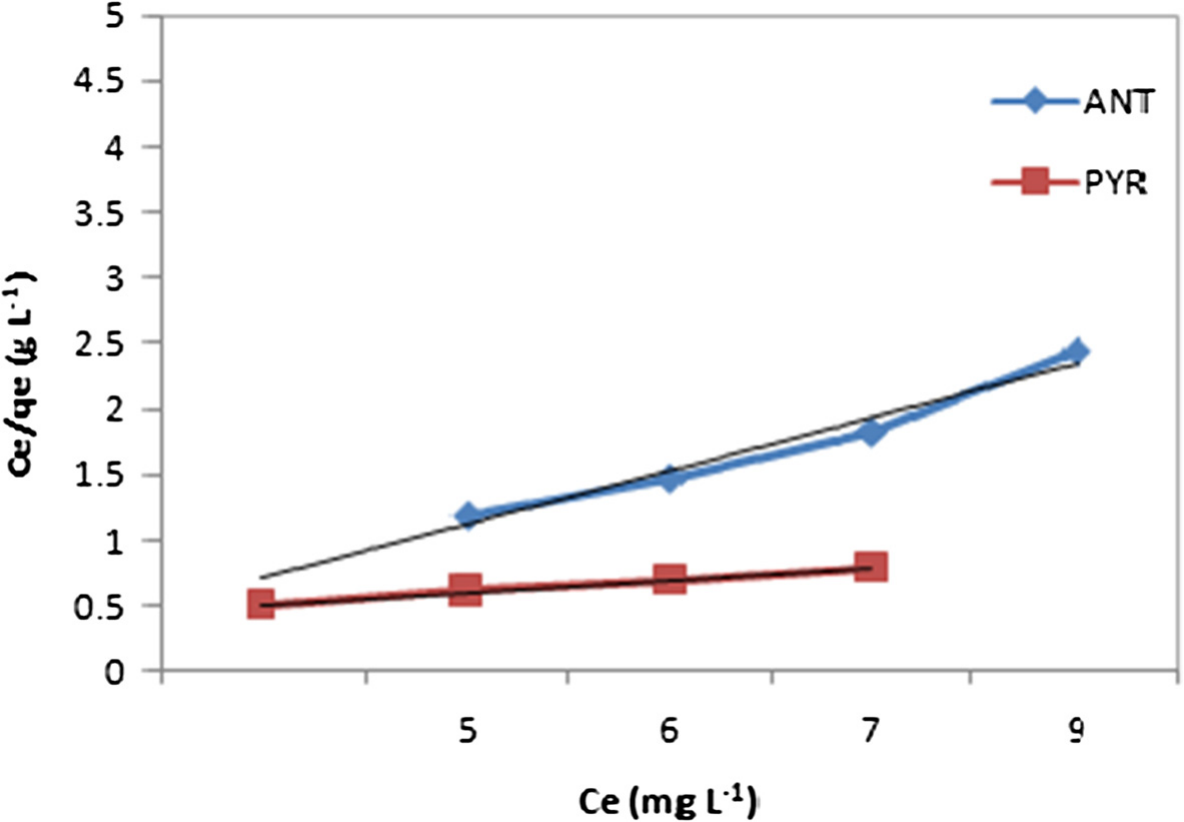
Langmuir isotherm for ANT/PYR adsorption onto CGMMNPs.

Langmuir is the most widely used adsorption isotherm. Langmuir adsorption isotherms have been used to determine the capacity of a sorbent by determining the amount of PAHs adsorbed by one gram of sorbent (CGMMNPs). A plot of Ce/qe versus Ce (Figure 10) shows a liner relationship with the slope of 1/q_max_. The values q_max_ and K_L_ can be estimated as 4.168 mg/mg and 0.98 L/mg, from the slope and intercept respectively, these are shown in Table 1.

**Table 1:** Isotherm parameters obtained by the linear method

**Langmuir isotherm model**				
PAH	Temperature	qmax (mg g^−1^)	K_L_(L mg-^2^)	R^2^
ANT	20°C	2.541	0.756	0.968
PYR	20°C	10.560	4.351	0.998
**Frendlich isotherm model**				
PAH	Temperature	K, (mg g^−1^)(L mg^−1^)^1/nc^	n	R^2^
ANT	20°C	2.718	4.472	0.992
PYR	20°C	3.458	4.478	0.990
**Temkin isotherm model**				
PAH	Temperature	A(Lg^−1^)	B (J mol^−1^)	R^2^
ANT	20°C	225.879	0.235	0.994
PYR	20°C	16.643	0.243	0.990

**Figure 11:**
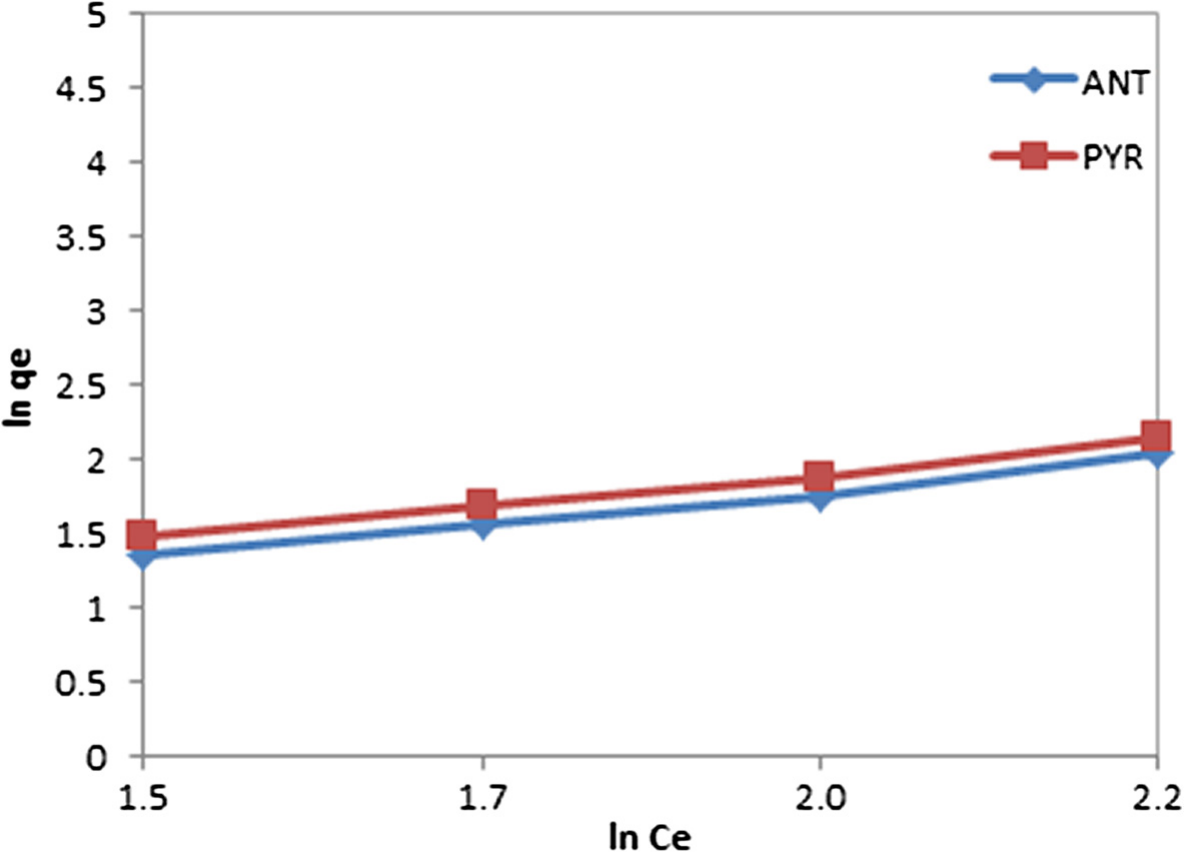
Freundlich isotherm ANT/PYR adsorption onto CGMMNPs.

The Freundlich adsorption isotherm represents the amount of PAH adsorbed per unit of adsorbent. From a plot of ln qe (mg/g) versus ln Ce (mg/L) (Figure 11), evaluations for slope and intercept can be calculated, as presented in Table 1. A linear form of a plot indicates applicability of the classical adsorption isotherm in this system.

**Figure 12:**
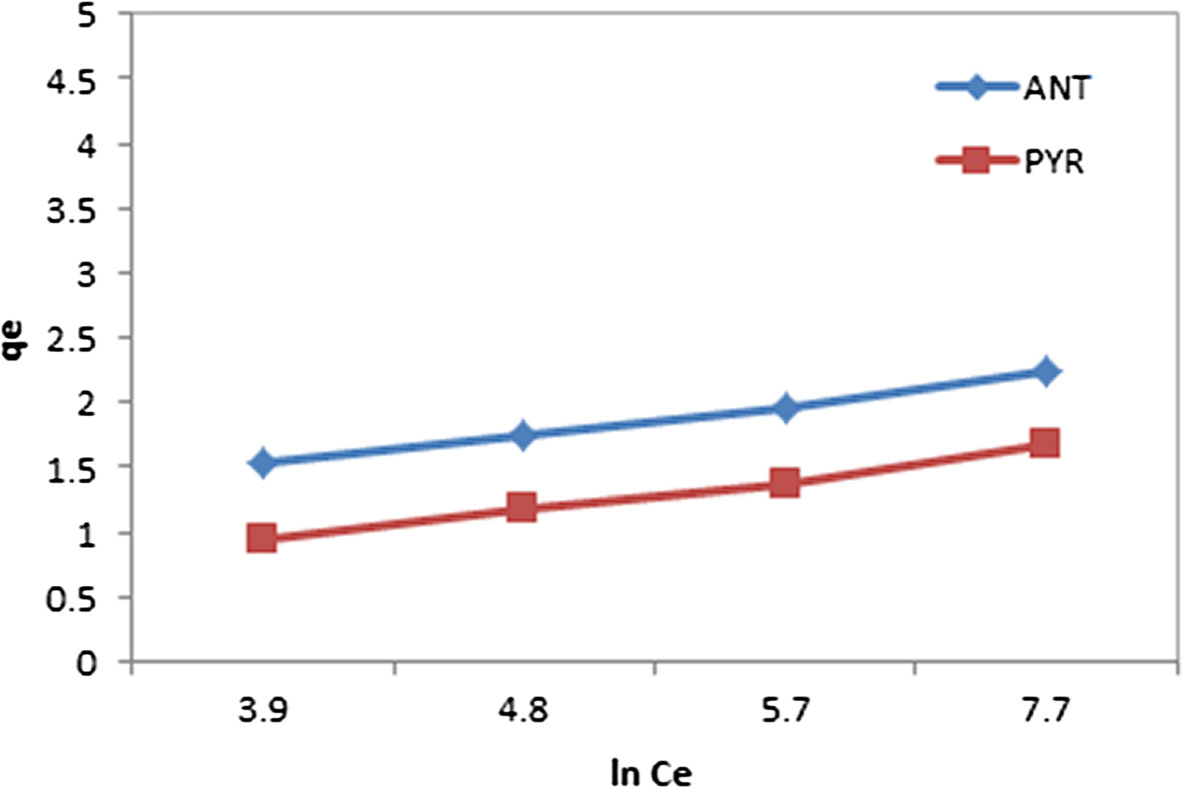
Temkin isotherm ANT/PYR adsorption onto CGMMNPs.

The Temkin equation suggests a linear decrease of sorption energy as the degree of completion of the adsorption sites of adsorbent is increased. Plotting qe versus ln Ce enables determination of the constants A and B (see Figure 12 and Table 1).

### Application of the method

CGMMNPs were used to determine PAHs (ANT, PYR) in water from Anzali marsh, Gilan state, Iran. pH level of the water sample was adjusted to the optimum pH. Adsorption with CGMMNPs coupled with HPLC was applied to determine PAHs in the water sample. Since no ANT or PYR was detected in the water sample, 100 mL water sample was spiked with 0.02 and 0.04 mg of ANT, PYR before applying the procedure. The results indicated applicability of the process for PAHs determination with high recovery (>90%). Experiments show that the method can be successfully applied for determination of PAHs in a sample of water from the environment.

## Conclusion

A method for the graft polymerization of MNPs and subsequently coupling it with aromatic compound as a novel adsorbent was introduced. The nano adsorbent had good potential for the fast removal of PAHs from large volume samples. The nano adsorbent also demonstrates the advantages of high adsorption capacity and high chemical stability. On the basis of Langmuir isotherm analysis, the monolayer adsorption capacity was determined as 4.17(mg/g) at 20°C.

## Competing interests

The authors declare that they have no competing interests.

## Authors’ contributions

The authors appreciate University of Tehran for providing financial and instrumental support to conduct this work. All authors read and approved the final manuscript.
